# Graded, multidimensional intra- and intergroup variations in primary progressive aphasia and post-stroke aphasia

**DOI:** 10.1093/brain/awaa245

**Published:** 2020-09-17

**Authors:** Ruth U Ingram, Ajay D Halai, Gorana Pobric, Seyed Sajjadi, Karalyn Patterson, Matthew A Lambon Ralph

**Affiliations:** a1 Division of Neuroscience and Experimental Psychology, School of Biological Sciences, University of Manchester, UK; a2 MRC Cognition and Brain Sciences Unit, University of Cambridge, Cambridge, UK; a3 Department of Neurology, University of California, Irvine, Irvine, USA; a4 Department of Clinical Neurosciences, University of Cambridge, Cambridge, UK

**Keywords:** aphasia, stroke, neurodegeneration, classification

## Abstract

Language impairments caused by stroke (post-stroke aphasia, PSA) and neurodegeneration (primary progressive aphasia, PPA) have overlapping symptomatology, nomenclature and are classically divided into categorical subtypes. Surprisingly, PPA and PSA have rarely been directly compared in detail. Rather, previous studies have compared certain subtypes (e.g. semantic variants) or have focused on a specific cognitive/linguistic task (e.g. reading). This study assessed a large range of linguistic and cognitive tasks across the full spectra of PSA and PPA. We applied varimax-rotated principal component analysis to explore the underlying structure of the variance in the assessment scores. Similar phonological, semantic and fluency-related components were found for PSA and PPA. A combined principal component analysis across the two aetiologies revealed graded intra- and intergroup variations on all four extracted components. Classification analysis was used to test, formally, whether there were any categorical boundaries for any subtypes of PPA or PSA. Semantic dementia formed a true diagnostic category (i.e. within group homogeneity and distinct between-group differences), whereas there was considerable overlap and graded variations within and between other subtypes of PPA and PSA. These results suggest that (i) a multidimensional rather than categorical classification system may be a better conceptualization of aphasia from both causes; and (ii) despite the very different types of pathology, these broad classes of aphasia have considerable features in common.

## Teaser

Language impairments caused by stroke and by neurodegeneration (primary progressive aphasia) have overlapping symptomatology and nomenclature but have rarely been compared in detail. Ingram *et al.* show that the two have considerable features in common and the variations of phenotype reflect four graded behavioural dimensions.

## Introduction

Aphasia is an impairment of the ability to comprehend and formulate language following acquired brain damage, which manifests as difficulties across multiple modalities of language use (e.g. reading, auditory comprehension, expressive language) (Rosenbek *et al.*, 1989). Causes of acquired brain damage leading to aphasia include stroke and neurodegeneration. The latter cause results in a form of aphasia termed primary progressive aphasia (PPA) (Mesulam, 2001). To differentiate the two, we will refer to aphasia as a consequence of stroke as post-stroke aphasia (PSA). Two clinical and theoretical issues are addressed in this study. First, despite the similarity of symptoms and nomenclature in subtypes of PSA and PPA, there have been few—if any—detailed direct comparisons across the full range of PSA and PPA. Second, although diagnostic subtypes have been proposed for both forms of aphasia, patients often vary greatly within each category or commonly fall between classifications (and thus are referred to as ‘mixed’). This suggests that the phenotype differences observed across patients might reflect graded variations across multidimensional aphasic spectra rather than a series of mutually exclusive, coherent diagnostic categories (Lambon Ralph *et al.*, 2003; Stopford *et al*., 2008; Migliaccio *et al*., 2009; Ridgway *et al*., 2012; Warren *et al*., 2012). By combining detailed assessment data across the full ranges of PSA and PPA, this study was able to map out these graded inter- and intragroup variations.

Although arising from very different pathologies, PSA and PPA share symptomatology. Despite these clear superficial behavioural similarities, detailed direct comparisons between PSA and PPA are rare and thus it is still unclear if the degree and nature of the symptoms are the same, or if the vocabulary terms used to describe the patients and their symptoms are truly equivalent. The small number of previous comparative studies have been focused on either specific tasks or linguistic/cognitive domains. For example, Patterson *et al.* (2006) compared speech production and phonological deficits in a selection of non-fluent subtypes of PSA and PPA. Jefferies and Lambon Ralph (2006) compared semantically impaired PSA and PPA patients on a range of linguistic and non-linguistic semantic tasks (Jefferies *et al.*, 2008). Thompson *et al.* (2013) compared syntactic processing in agrammatic and anomic forms of PSA and PPA (see also Budd *et al.*, 2010; Thompson *et al.*, 2012; Faria *et al.*, 2013). Mesulam *et al.* (2015) compared comprehension of words and sentences in different subtypes of PPA, splitting the cases according to whether their atrophy encroached ‘Wernicke’s area’—a region that post-stroke aphasia classically implicates in comprehension deficits (*cf*. Wernicke’s aphasia). Whilst these important studies have advanced our understanding of specific language features for selected subtypes of PPA/PSA, larger scale studies are needed for at least two reasons: (i) it is important to explore performance simultaneously across a broad spectrum of language and cognitive areas in order to situate and understand any one specific task; and (ii) comparisons of select PPA/PSA subtypes make the assumption that the subtypes can be readily identified and are the most appropriate basis for the comparison.

Individuals with PPA or PSA display considerable variation in the nature and severity of their impairments (e.g. naming, repetition, comprehension, reading, etc.)—but what is the basis of these variations? To clinical and research professionals working with patients with aphasia, it is clear that there is an underlying structure in their aphasic performance (i.e. heterogeneity in aphasia phenotype is not caused by random variation and noise). Ruling out random variation, behavioural variation in health or disease can be split into two types reflecting the presence of either multiple, mutually exclusive coherent categories of person/patient, or graded variations along different dimensions. All true categorical classification systems are based on two assumptions: (i) that there is homogeneity within each category or type; and (ii) that there are distinct boundaries between categories (Schwartz, 1984).

As is traditional in neurology and neuropsychology, categorical subtypes of PSA and PPA have been proposed. The Boston Diagnostic Aphasia Examination (BDAE) (Kaplan, 1983), for example, categorizes PSA patients into one of seven subtypes based on their relative strengths and weaknesses in repetition, speech output fluency and comprehension. In addition to this, the BDAE also uses this information to give an indication of the level of impairment within each subtype. The Western Aphasia Battery (WAB) (Kertesz, 2007) categorizes patients into discrete subtypes and provides the aphasia quotient to give a sense of general aphasia severity regardless of subtype. Likewise, the consensus derived classification system for PPA (Gorno-Tempini *et al.*, 2011) delineates three categorical subtypes: semantic dementia/semantic variant PPA, non-fluent/agrammatic variant PPA and logopenic variant PPA (lvPPA), though numerous additional subtypes are often proposed [such as agrammatic PPA without apraxia of speech (Tetzloff *et al.*, 2019), or primary progressive apraxia of speech (Josephs *et al.*, 2012)]. There is evidence, however, that a strict categorical approach is limited and does not capture the true nature of the patients’ variations. Thus, (i) rather than homogeneity within each category, there is significant variation (e.g. consider the different presentations of anomic aphasia or non-fluent progressive aphasia); (ii) patients’ categorical membership can change (with recovery in PSA and decline in PPA); and (iii) there are blurred boundaries between categories (e.g. the boundary between conduction aphasia and Wernicke’s aphasia). One consequence is that in both PSA and PPA there is a considerable proportion of patients who must be classified as having ‘mixed’ aphasia because they either do not fulfil the criteria for any subtype, or even fulfil the criteria for more than one subtype (Benson, 1979; Wertz *et al.*, 1984; Mesulam *et al.*, 2008, 2012; Knibb *et al.*, 2009; Sajjadi *et al.*, 2012*a*; Gil-Navarro *et al.*, 2013; Harris *et al.*, 2013; Matias-Guiu *et al.*, 2014; Wicklund *et al.*, 2014; Botha *et al.*, 2015; Spinelli *et al.*, 2017; Utianski *et al.*, 2019).

These limitations of the categorical approach implied by the syndrome classification systems have long been understood clinically (Caramazza, 1984) (e.g. the limited use of broad ‘brush stroke’ classifications like Broca’s aphasia in describing an individual’s unique impairments for the purpose of therapy) (Feyereisen *et al.*, 1986; Gordon, 1998). In PSA, the use of the subtype labels from the BDAE/WAB classification systems has evolved over time and now they are often used in sophisticated and flexible ways. The labels have come to refer to the heterogeneous constellation of strengths and weaknesses making up a particular individual’s aphasic profile, rather than to imply that the patient fits the exact diagnostic criteria for a specific aphasia subtype. It seems likely that this natural evolution in the use of these labels is very important and might tell us something important about the variation in the patients’ presentations, paralleling our argument here that patients vary along graded dimensions.

This hypothesis arises from formal explorations of an alternative, non-categorical way to conceptualize behavioural variations (Butler *et al.*, 2014; Mirman *et al.*, 2015*a*). These new approaches are based on the second source of individual differences noted above—namely, graded variations along continuous behavioural dimensions. Recent studies have reconceptualized the variations in PSA as forming an aphasic multidimensional space with each patient taking up a different position (typically varying in terms of phonology, semantics, speech fluency and, when assessed, non-language cognitive skills) (Butler *et al.*, 2014; Halai *et al.*, 2017, 2018*a*; Schumacher *et al.*, 2019). In this formulation, the classical aphasia labels (e.g. conduction, Broca’s, etc.) do not represent categories *per se*, but rather are verbal pointers to a subregion in the multidimensional space. By way of analogy, one can think of patients as colour hues across the red, green and blue (RGB) colour space. It is possible to recognize clear differences [such as yellow (e.g. Broca) versus blue (Wernicke), etc.] but also to capture the graded and unbounded variations between colours [e.g. there are many types of blue, its boundary with greens or violets is unclear, there are many hues (e.g. teal, maroon, etc.) that are hard to classify uniquely, and perceivers (*cf*. clinicians/researchers) have slightly different definitions for each colour (*cf*. clinical label)].

Accordingly, some key aims of the current study were: (i) to test if the same approach can be applied to PPA (in contrast to other studies where methods capable of capturing graded variation have only been used as an intermediate step towards categorizing proposed subtypes of PPA) (Mesulam *et al.*, 2009; Hoffman *et al.*, 2017); (ii) to compare the multidimensional spaces for PSA and PPA; and (iii) to test if a single multidimensional space can be formed for PSA and PPA to allow direct, intra- and intergroup comparisons. Importantly, the aim of situating PSA and PPA in a shared multidimensional space was not to differentiate between these aetiologies. Rather, we used this shared space as a platform for a larger-scale direct comparison without selecting specific subtypes or cognitive/linguistic processes (as mentioned above). These aims were tackled through two large PSA and PPA cohorts (inclusive of typical and mixed cases), both completing large-scale, detailed neuropsychological and aphasiological test batteries.

## Materials and methods

We initially applied principal component analysis (PCA) to PPA and PSA separately. This allowed us to compare qualitatively the resultant multidimensional space for each patient group without forcing the two groups into a single space. Given that the two group-specific PCA results were similar in form, available PSA patients were reassessed using a shared test battery derived from the PPA test battery, so that all patients could be entered simultaneously into a unified PCA. This enabled direct comparisons of both intergroup and intragroup variations.

### Patients

All patients were recruited non-selectively (with respect to subtype-level behavioural presentation) to sample the full space and severities of behavioural impairments in both PPA and PSA. Although diagnostic subtype labels were applied for descriptive purposes, the inability to apply a single diagnostic label was not grounds for exclusion in either cohort. Demographic details are shown in Table 1.

**Table 1:** Demographic details per subtype of the PSA and PPA cohorts

Group	Subtype	*n* (F)	Age	Education, years	Time with aphasia, years
PSA	Anomia	30 (11)	63.8 (13.4)	12.4 (2.7)	4.2 (4.3)
	Broca	13 (1)	62.7 (13.0)	11.9 (1.8)	4.3 (3.4)
	Conduction	4 (1)	62.0 (10.7)	13.8 (3.2)	1.8 (0.9)
	Global	9 (0)	66.4 (9.0)	11.3 (0.7)	5.9 (4.6)
	Mixed non-fluent	16 (4)	68.1 (9.0)	11.5 (1.0)	6.3 (4.9)
	TMA	2 (1)	74.5 (2.1)	11.0 (0.0)	6.8 (4.1)
	TSA	1 (0)	63.0	12.0	2.0
	Wernicke/conduction	1 (1)	77.0	16.0	2.8
PPA	Logopenic	2 (1)	71.0 (4.2)	11.0 (2.8)	2.0 (0.0)
	Mixed PPA	16 (12)	72.7 (5.2)	10.8 (1.9)	3.3 (1.4)
	PNFA	12 (7)	69.3 (7.3)	13.0 (3.8)	3.2 (1.4)
	Semantic dementia	16 (8)	67.1 (8.5)	13.9 (3.3)	4.2 (1.3)

Data are presented as mean (standard deviation). TMA = transcortical motor aphasia; TSA = transcortical sensory aphasia.

Seventy-six patients with chronic PSA were prospectively recruited from community groups and speech and language therapy services in the North West of England. Patients were included if they reported a single left hemisphere stroke at least 12 months prior to assessment and were native English speakers. A portion of the PSA cases have been reported by Butler *et al.* (2014) and Halai *et al.* (2017) (31/70), and by Halai *et al.* (2018*b*) (70/76). All patients were classified into diagnostic subtypes by application of the BDAE (Kaplan, 1983). All patients provided informed consent under approval from the North West Multi-Centre Research Ethics Committee, UK. Thirty-four of the 76 PSA cases were available for retesting on the shared battery for the unified PCA on PSA and PPA. Of these 34 cases, 15 had anomic aphasia, five had Broca’s aphasia, three had conduction aphasia, five had global aphasia, five were classified as mixed non-fluent aphasia, and one had transcortical motor aphasia.

Forty-six patients with PPA were prospectively recruited from memory clinics at Addenbrooke’s Hospital, University of Cambridge (UK), as part of a longitudinal study of PPA (Sajjadi, 2013). These cases have previously been reported by Sajjadi *et al.* (2012*a*,[Bibr awaa245-B65]*b*, *c*, 2014) and Hoffman *et al.* (2017). Patients with PPA were recruited based on meeting the core criteria for PPA (Mesulam, 2001) then classified into a diagnostic subtype by application of the Gorno-Tempini *et al.* (2011) criteria, or given the label ‘mixed PPA’ if unclassifiable (see Sajjadi *et al.*, 2012*a* for details of how cases were diagnosed and reasons for being unclassifiable). In brief, a collection of tests was chosen to measure all the consensus-proposed language features of the PPA subtypes. A score 1.5 standard deviations (SD) below control norms was considered impaired. A large proportion of the cohort was classified as semantic dementia, progressive non-fluent aphasia (PNFA) or mixed PPA (mPPA). We note that in this study only a very small number met the criteria for lvPPA. Future studies are needed to explore how a larger sample of lvPPA distributes across the shared PCA-derived multidimensional space. Exclusion criteria included other causes of aphasia (e.g. non-neurodegenerative pathology), non-native English speakers and any other neurological or major psychiatric illness. The PPA dataset comprised data from two longitudinal rounds of testing to assess change over time. On average, the second round of data was collected after 12.7 months (SD: 0.9 months). Following Lambon Ralph *et al.* (2003), the participants who had scores for both rounds were treated as pseudo-independent observations. This resulted in a total of 82 data-points for analysis. Of these 82 observations, 26 were mPPA, 24 were PNFA, 28 were semantic dementia and four were lvPPA. This approach was used because PCA is a data-hungry method (Guadagnoli and Velicer, 1988) that benefits from having adequate sampling of as much of the potential PPA ‘space’ as possible. All patients, or next of kin where appropriate, provided informed consent under approval from the Cambridge Regional Ethics Committee.

### Neuropsychological assessments

#### Post-stroke aphasia test battery

The tests included in the PSA test battery are shown in Supplementary Fig. 1, and described in Halai *et al.* (2017) and Butler *et al.* (2014). Briefly, the battery assessed connected speech, comprehension of grammar, auditory discrimination, repetition, semantic knowledge, naming, working memory and attention/executive function.

#### Primary progressive aphasia test battery

The tests included in the PPA test battery are shown in Supplementary Fig. 2, and described by Sajjadi (2013). Briefly, this battery assessed connected speech, comprehension of grammar, grammatical ability in sentence production, repetition, semantic knowledge, naming, phonological discrimination, working memory, attention and executive function, visuospatial skills, and oro-buccal and limb praxis.

#### Shared battery

To establish the shared multidimensional space of PSA and PPA, available PSA cases were re-tested on a shared test battery, which was derived from the PPA test battery. Thirty-three tests were including in the shared battery, shown in Supplementary Fig. 3. This battery assessed attention and executive function, repetition, sentence comprehension and production, semantic memory, visuospatial skills, praxis, connected speech, naming, and phonological discrimination.

### Data analysis

All raw behavioural scores were converted to percentages. For measures without a fixed maximum score, scores were converted to a percentage of the maximum score across the relevant cohort or both cohorts for the unified PCA. Missing data were imputed using probabilistic principal component analysis (PPCA) (Ilin and Raiko, 2010). This approach was chosen as the results were stable when compared to versions of the analyses without imputation (i.e. list-wise exclusion analysis). PPCA requires that the number of components to be extracted is specified *a priori*, so a k-fold cross validation approach (Ballabio, 2015) was used to choose the number of components giving the lowest root mean squared error for held-out cases over 1000 permutations. This approach was also used to select the optimal number of components for subsequent PCA using the imputed dataset. The imputed datasets were entered into PCAs (conducted in SPSS 23), with varimax rotation to aid cognitive interpretation of the extracted dimensions. This interpretation was based on the core aspects of the tests with the largest loadings onto each factor. The factor labels necessarily capture less information than the test loadings (which are provided in the Supplementary material) but serve as a useful shorthand. The adequacy of the sample size for each PCA was determined using Kaiser-Meyer-Olkin measure of sampling adequacy.

The separate PCAs for PPA and PSA could not be compared directly as they did not share the same tasks, so they were compared qualitatively by analysing the type of tasks that loaded most heavily onto each extracted component. This approach was also used to compare the separate PCAs to the unified PCA. However, since the unified PCA was conducted on data from both groups on the shared battery, this made direct, quantitative, intra- and intergroup comparisons possible. To put the relative regions of the multidimensional space occupied by PSA and PPA into perspective, control norms were projected into the unified PCA space by normalizing to the patient mean and SD, and then using the factor coefficients to generate factor scores for an average control participant. Subtype classifications of PSA and PPA were derived separately, using tests included in the different battery for each aetiology (e.g. sentence repetition assessed with different sentences in the BDAE versus the repetition task used by Sajjadi *et al*., 2012*a*). We avoided the problems of trying to make quantitative comparisons between the aetiologies via different test batteries, by conducting intergroup comparisons using the shared battery, and by comparing individuals’ positions in the multidimensional space regardless of their subtype categorization.

Finally, formal analyses were conducted to test for the presence of subgroup categories (i.e. subgroups with relatively high intragroup homogeneity and distinct intergroup differences). The motivation for this analysis was as follows: working under the hypothesis that the structure of variation in PSA and PPA is driven by graded variation along multiple dimensions means that methods like cluster analysis (e.g. k-means clustering) would be inappropriate for detecting potential graded variation. Yet, visual inspection of the scatter plots defined by the extracted PCA dimensions (Fig. 2) showed many regions of extensive overlap but also (for semantic dementia) some more uniquely occupied regions. Therefore, we sought to quantify this by conducting a form of data-driven classification analysis within the graded multidimensional space, rather than using formal cluster analysis. We note that the principal dimensions were not intended as a new way to categorize patients. Instead, we took it to be the case that if one or more subgroups formed a true category, they would be represented in the PCA multidimensional space as a homogenous group of data-points, and it would be possible to define formal diagnostic boundaries with the other subgroups in terms of cut-off scores on each extracted dimension.

To investigate this, formally, the unified PCA was systematically swept to find the combination of cut-off values across all dimensions that gave the highest sensitivity index (*d* prime, *d*′) value per diagnostic subtype. Crucially, the calculations of sensitivity were only within aetiology, i.e. only considering subtypes from the same cohort; the aim of this analysis was not to differentiate PSA and PPA. PCA solutions are always scaled using *z*-scoring, and in this study the dimensions ranged from approximately −3 to +2 with zero representing the centroid of the patient cohort. These dimensions were swept iteratively at intervals of 0.05. The *d*′ equation was adapted to account for extreme values (0 or 1) for the rate of hits or false alarms (Macmillan and Kaplan, 1985), resulting in the maximum *d*′ value achievable being 4.65; thus a *d*′-value near 4.65 would be suggestive of distinct categorical boundaries and within-group homogeneity. To establish the likelihood of achieving these *d*′-values by chance, diagnostic group membership was randomized within aetiology and *d*′ recalculated over 10 000 iterations to give a distribution of *d*′-values.

The combination of cut-off values along each dimension that gave the maximum *d*′-value (i.e. highest possible sensitivity) were then treated as diagnostic ‘criteria’ for new data-driven diagnostic groups. The hits from the *d*′ analysis represent cases whose factor scores correctly met the cut-off values for their own data-driven diagnostic group. The false alarms represent cases whose factor scores incorrectly met the cut-off values for any other data-driven diagnostic group, i.e. cases who were misclassified. These misclassifications occurred despite the cut-offs representing the best possible (highest sensitivity) between-group boundaries that could be found in the iterative sweep through the entire multidimensional space. The distribution of misclassifications amongst subtypes of each aetiology was extracted from the false alarms associated with each data-driven diagnostic group. It is important to note that it was possible for a single case’s factor scores to meet the cut-off values for more than one data-driven diagnostic group simultaneously (or none), and this may or may not have included their own group.

### Data availability

Anonymized data are available on reasonable request for academic (non-commercial) purposes, although restrictions may apply to adhere to participant consent and anonymity.

## Results

### Principal component analysis

#### Post-stroke aphasia

The PCA for the PSA cohort was robust (Kaiser-Meyer-Olkin = 0.84) and produced a four-factor rotated solution that accounted for 76.7% of variance in PSA patients’ performance (F1 = 30.4%, F2 = 17.5%, F3 = 15.2%, F4 = 13.7%). The factor loadings of each behavioural assessment onto the extracted components are shown in Supplementary Fig. 1.

Measures loading heavily onto the first factor were tests of repetition [Psycholinguistic Assessments of Language Processing in Aphasia (PALPA) words, non-words], naming (Boston, Cambridge), phonological working memory (digit span), auditory comprehension [Comprehensive Aphasia Test (CAT) sentence comprehension], and phonological sensitivity (PALPA minimal pairs). These tests all require phonological processing; hence we called this factor ‘Phonology’. The strong loadings from the naming tests onto this factor are likely to be driven by the fact that many of the cases in this PSA cohort have a core phonological processing impairment (hence this phonology factor explained the greatest amount of variance in the PCA) and so the phonological aspect of naming is compromised in these patients.

The second factor had strong loadings from the two measures designed to assess attention and executive function (Brixton, Raven’s). Other tests not designed to measure executive function *per se* also had strong loadings onto this factor (e.g. minimal pairs, spoken word-to-picture matching, etc.). This probably reflects the fact that tasks designed to assess various language activities also call upon generalized attention and executive skills (e.g. to compare verbal stimuli, decide between responses, etc.). This is true for the semantic tests (aligning with the fact that semantic cognition requires access to semantic representation but also executively-related processes (Jefferies and Lambon Ralph, 2006; Thompson *et al.*, 2018) and also with respect to the working memory, abstract reasoning and problem-solving requirements in other language tests (e.g. sentence comprehension and minimal pairs). The nature of PCA means that it decomposes and orthogonalizes these sources of variation. As a result, individual tests can have strong loadings across multiple extracted factors and each factor points towards a shared underpinning process. Thus, for this second factor, whilst spanning different aspects of language and cognition, these tests all share the feature of requiring attentional or executive processing skills; hence, we called this factor ‘Executive Function’.

Measures with strong loadings onto the third factor included speech rate (words per minute) and speech quanta (total number of words). We called this factor ‘Speech Fluency’. We note that the Camel and Cactus test, an executively demanding test of semantic associative relationships, also had a very strong loading onto this factor. This result is surprising and has not occurred in our previous investigations, where it has loaded onto the executive and semantic factors (Butler *et al.*, 2014; Halai *et al.*, 2017). We could speculate that this result might reflect variation in another form of executive process (distinct from the executive process that seems to be captured by the second factor), which might be involved in (i) iteratively generating and assessing semantic associations (Camel and Cactus and synonym judgement); (ii) generating speech (words per minute and total words produced); and (iii) generating and monitoring ‘chunks’ to complete the backwards digit span task. Indeed, Schumacher *et al.* (2019) found that the Camel and Cactus test loaded onto an ‘Inhibit-Generate’ executive component. However, without more measures of attention and executive function it is not possible to test these speculations. We chose to give this third factor the subjective label of ‘Speech Fluency’ given that the strongest loadings are from words per minute and total number of words.

Measures with strong loadings onto the fourth factor were tests of semantic knowledge (synonym judgement, word-to-picture matching) and semantic richness of speech (mean length per utterance). Furthermore, measures of naming (Boston, Cambridge) and sentence comprehension (CAT) also had moderate factor loadings onto this factor. These tests all require semantic knowledge; hence we called this factor ‘Semantics’.

#### Primary progressive aphasia

The PCA for the PPA cohort also generated a robust result (Kaiser-Meyer-Olkin = 0.85) with a five-factor rotated solution, which accounted for 72.4% of variance (F1 = 23.7%, F2 = 17.8%, F3 = 14.5%, F4 = 9.8%, F5 = 6.7%). The factor loadings of each behavioural assessment onto the extracted components are shown in Supplementary Fig. 2.

The tests loading onto the first factor all required retaining phonological information (e.g. single digits, words, numbers, whole sentences) in mind; hence we called this factor ‘Phonological Working Memory’. These measures included tests of phonological sensitivity (non-word minimal pairs), attention and executive function (digit span forwards and backwards, letter span similar and dissimilar phonemes), repetition (words, non-words and sentences), sentence comprehension [SECT, Test for Reception of Grammar (TROG)] and cube counting (Visual Object and Space Perception, VOSP).

The second factor comprised heavy loadings from tests relying on semantic knowledge; hence we called this factor ‘Semantics’. These tasks included tests of semantic knowledge (Cambridge naming, Point from Repeat and Point, Category Fluency), semantic association (Camel and Cactus), recognition of irregular words, and sentence comprehension (SECTV).

The third factor was characterized by strong loadings from measures of speech rate (words per minute) and speech quanta (total number of words), in addition to oro-buccal praxis. A test of executive function requiring drawing and counting (Trail Making Test, TMT-A) also had high loadings (note, patients often count under their breath or out loud to complete the TMT-A). Accordingly, this factor appeared to capture the motor aspect of speech, hence we called this factor ‘Motor Speech Production’.

Visuospatial tests of executive function loaded heavily onto the fourth factor. Specifically, tests of switching (TMT-B), counting and visual imagery (VOSP), and copying and visuospatial recall (Rey Complex Figure) had high loadings on this factor, hence we called this factor ‘Visuo-Executive Function’.

Loadings onto the fifth factor were dominated by tests of sentence production (Mississippi Aphasia Screening Test, MAST), measures of semantic richness of speech (mean length per utterance) and generation of items (letter fluency from the Addenbrooke’s Cognitive Examination – Revised) (Mioshi *et al.*, 2006). Having accounted for motor speech production, semantics and executive demands in earlier factors, the remaining aspect of these tests which might be captured in this final independent factor could be the generative aspect of speech production. Hence, we called this factor ‘Speech Generation’.

#### Unified principal components analysis on the shared battery

Given that the two group-specific batteries and PCAs generated similar types of dimensions (phonology, semantics, executive skill and aspects of speech production), a formal direct comparison through a shared battery and single PCA spanning both groups was both merited (i.e. there was *prima facia* evidence of shared symptoms and variations) and permitted formal intra- and intergroup comparisons by enabling inclusion of all patients across a single multidimensional space. The unified PCA was again robust (Kaiser-Meyer-Olkin = 0.88), with a four-factor rotated solution accounting for 67.4% of variance in patient performance (F1 = 23.5%, F2 = 16.6%, F3 = 14.8%, F4 = 12.6%), and bore a strong relationship with the factors identified in the group-specific test batteries. The factor loadings of each behavioural assessment onto the extracted components are shown in Supplementary Fig. 3.

The first factor had high loadings from tests of repetition (words, non-words, sentences), phonological sensitivity and attention (digit and letter spans, non-word minimal pairs), and sentence comprehension (auditory and visual). These tests all require intact phonological processing, hence we called this factor ‘Phonology’.

There were high loadings on the second factor for tests of semantic knowledge (Cambridge naming, pointing from the Repeat and Point test), generation of items in a semantic category [category fluency from the Addenbrooke’s Cognitive Examination (ACE)], sentence comprehension (SECT) and address recall and recognition from the ACE. Hence, we called this factor ‘Semantics’.

Tests of attention and executive function in the visuospatial domain (VOSP, TMT, Rey Complex Figure) all loaded heavily onto the third factor. As above, we called this factor ‘Visuo-Executive Function’.

The fourth factor had high loadings from measures of speech quantity (words per minute, total number of words and mean length per utterance). Measures of praxis (oro-buccal and limb) and phonological working memory (digit span backwards) also had high loadings onto this factor. Given that phonological ability and executive functions have been accounted for already, this factor probably captured the speech production element of the digit span test (patients often repeat the string of digits to themselves before reporting them backwards). These tests therefore all require production of speech, and coupled with the loadings from the praxis tests, we interpreted this as a ‘Motor Speech Production’ factor.

The visuo-executive function and motor speech production factors had strong negative loadings from the TMT-B and TMT-A response times, respectively. We reran the analysis without these measures to ensure the result was stable and that the negative loadings were not an artefact of a coincidental correlation with general motor abilities. Pearson correlations between the original factors and their corresponding updated factors following the removal of the TMT response time measures were very high (F1 versus F1: 0.999, F2 versus F2: 0.997, F3 versus F3: 0.996, F4 versus F4: 0.991), showing that the PCA result was unchanged.

### Intergroup comparisons

**Figure 1:**
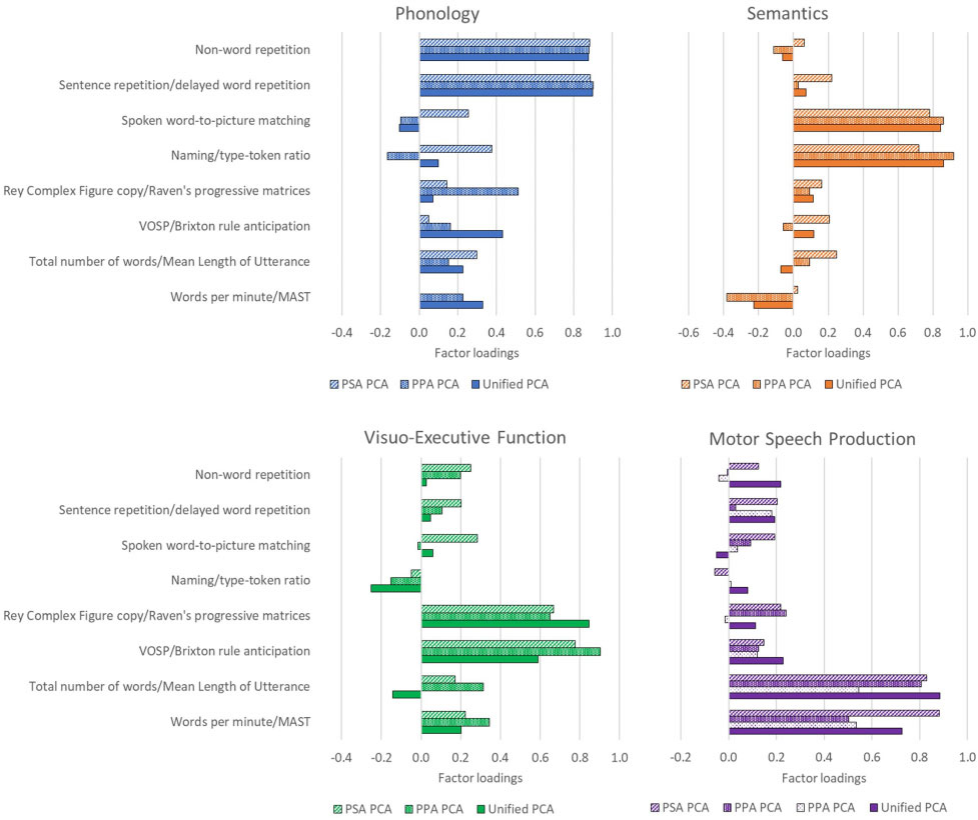
**Intergroup comparison of the underlying dimensions of variance in PSA and PPA.** Bars represent the factor loadings of exemplar tests onto each extracted factor. Factor loadings represent the weighting of each test on each factor and were used to suggest cognitive interpretations of the factors. The patterns of the bars represent the different PCAs; the PPA PCA extracted two speech production components, which are shown in different patterns on the motor speech production panel. MAST = Make a Sentence Test (Billette *et al*., 2015); VOSP = Visual Object and Space Perception battery (Warrington and James, 1991).

To illustrate the components extracted in each PCA, exemplar tests with strong and relatively unique loadings onto each factor across the three PCAs are plotted together in Fig. 1 (the full plots of all tests loadings on all PCA factors are shown in the Supplementary material). The specific example test chosen differed across PCAs due to the different test batteries, but where possible the same or a similar measure was chosen. Figure 1 highlights the similarity of the components extracted for both forms of aphasia, whether separately or in combination.

**Figure 2:**
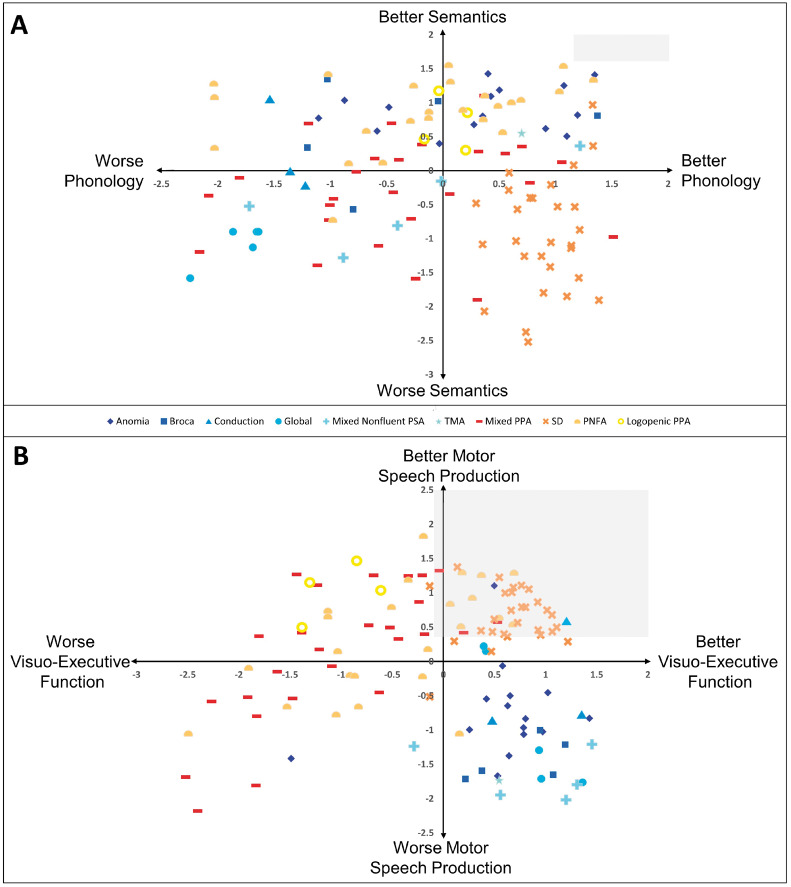
**Regions of the shared multidimensional space of PSA and PPA occupied by each diagnostic subtype.** Factor scores of all patients were plotted along all pairs of components extracted from the unified PCA. The origin is the mean of all patients. The factor scores are an expression of how many standard deviations a patient’s performance is from the group mean. The region of space reflecting preserved performance was calculated by projecting control norms into the patient space and is shaded in grey. PSA subtypes are blue-spectrum colours, PPA are red spectrum colours. SD = semantic dementia; TMA = transcortical motor aphasia.

Direct inter- and intragroup comparisons were possible in the shared multidimensional space of the unified PCA. Figure 2 plots the patients and their aphasia classifications into the 4D factor space (Fig. 2A maps the phonology and semantics factors, Fig. 2B speech production versus visuo-executive skill factors). Four key observations can be gleaned from these scatterplots: (i) intragroup graded differences: for both PPA (except semantic dementia, see below) and PSA there is considerable variation across cases within each subtype of aphasia and also overlap between the groups (e.g. conduction and anomic aphasia or PNFA and mPPA); (ii) intergroup differences: with regards to semantics and phonology the PSA and PPA cases are fully overlapping reflecting the clinical observation that the two aetiologies share many language symptoms; (iii) the two aetiologies are strongly separated in terms of speech fluency and co-occurring visuo-executive skills with the PSA cases dominating the space denoting poorer fluency yet better visuo-executive skills (Fig. 2B). All forms of PSA (even those referred to as ‘fluent’) were less fluent than the PPA patients (with the exception of the most severe PNFA and mixed cases), whilst only the semantic dementia subset were able to match the PSA on visuo-executive skills; and (iv) by eye, the only group that might form a coherent and separated cluster (*cf*. a true category) are those with semantic dementia in that they appear to uniquely occupy the combination of moderate-to-severe semantic impairment with good phonology (Fig. 2A) and good visuo-executive function and speech fluency (Fig. 2B). We tested formally whether semantic dementia and any other groups form a true category in the subsequent analysis.

### Intragroup graded variation

**Figure 3:**
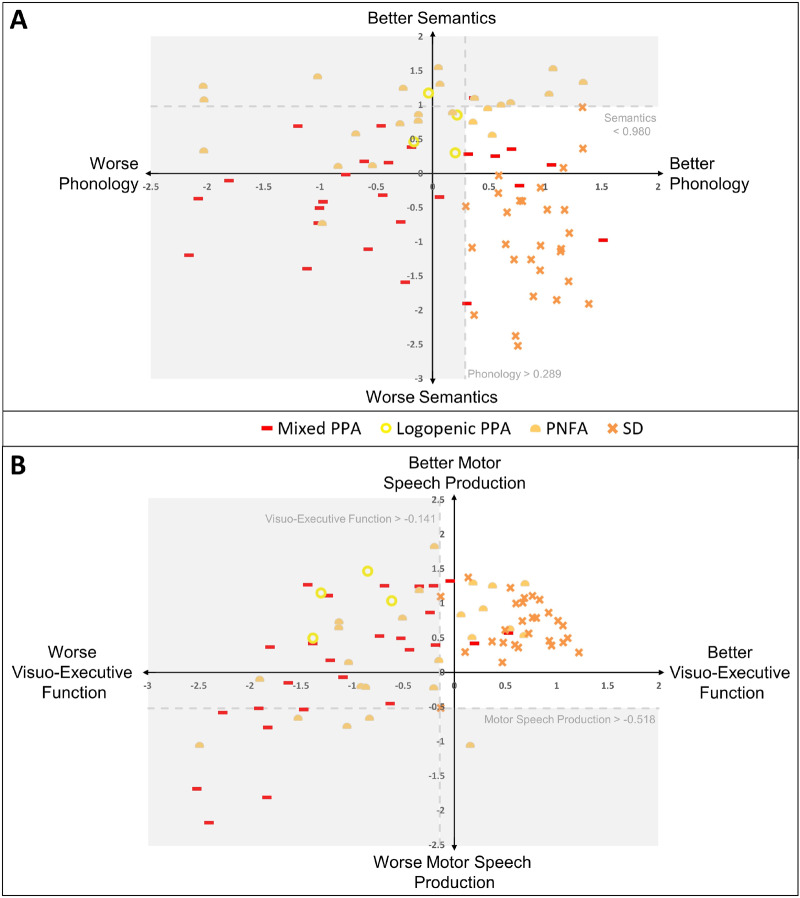
**Data-driven diagnostic cut-off values for semantic dementia.** Using a data-driven sweep at intervals of 0.05 through the entire four-dimensional space, the combination of cut-off values giving optimum sensitivity for semantic dementia was isolated. Applying the simultaneous combination of these four-dimensional cut**-**off values as diagnostic criteria (dashed lines) gave perfect selectivity for semantic dementia. This implies that semantic dementia shows within-group homogeneity and distinct between-group differences, suggestive of a true diagnostic category. This process was repeated for all subtypes of PPA and PSA within each aetiology (cut-off values and *d*′-values per subtype in Supplementary Table 1). SD = semantic dementia.

For each subtype within each aetiology, the best combination of ‘diagnostic’ values across all four dimensions was derived using a data-driven search (Supplementary Table 1). These values were treated as cut-offs defining new data-driven diagnostic groups, which were labelled according to the subtype from which the cut-offs were derived. An illustration of the data-driven diagnostic cut-offs for semantic dementia is shown in Fig. 3.

The pattern of hits and misclassifications associated with each combination of diagnostic cut-offs for PPA subtypes is shown in Table 2. The pattern of hits and misclassifications for PSA subtypes is shown in Supplementary Table 2, as these results will need validating in a larger cohort; in order to include a heterogeneous cohort reflecting the true phenotypic space of PSA, subtypes of PSA were included in this study even if they comprised only a single case. However, in terms of assessing whether the subtypes of PSA meet the assumptions of a true category, larger sample sizes will be needed to fully answer this question.

**Table 2:** Distribution of misclassifications between clinical and data-driven diagnostic PPA groups

Clinical diagnostic groups (*n*)	Data-driven diagnostic groups						
	Hits	Misclassifications					
		lvPPA	mPPA	PNFA	SD	None	>1
lvPPA (4)	100.0	–	50.0	100.0	0.0	0.0	100.0
mPPA (26)	92.3	0.0	–	38.5	0.0	3.8	34.6
PNFA (24)	91.7	4.2	20.8	–	0.0	4.2	16.7
Semantic dementia (28)	100.0	0.0	25.0	3.6	–	0.0	28.6

The cut-off values giving optimum sensitivity for each diagnostic group were treated as data-driven diagnostic criteria. Rows represent ‘real’ clinical diagnostic categories. The ‘Hits’ column represents the percentage of patients meeting the data-driven cut-off values for their own data-driven diagnostic group. The columns under ‘Misclassifications’ represent the percentage of cases whose factor scores (i) met the cut-off values for a different data-driven diagnostic group; (ii) did not meet the cut-off values for any of the data-driven diagnostic groups; and (iii) met the cut-off values for more than one data-driven diagnostic group. These ‘Misclassifications’ columns are not mutually exclusive, so row totals do not add up to 100%.

The data presented in Table 2 are the percentages of patients, from each clinical diagnostic subgroup of PPA, whose factor scores met the data-derived cut-off values for each diagnostic group. The rows represent the ‘real’ clinical diagnostic categories for patients in this study. The ‘Hits’ column represents the percentage of patients meeting the data-driven cut-off values for their own diagnostic group. The columns under ‘Misclassifications’ represent the percentage of cases whose factor scores (i) met the cut-off values for a different (i.e. incorrect) diagnostic group; (ii) did not meet the cut-off values for any of the possible data-driven diagnostic groups; and (iii) met the cut-off values for more than one data-driven diagnostic group (e.g. their own group and one other group). These ‘misclassifications’ are not mutually exclusive and thus cases falling into more than one classification are tabulated in the ‘>1’ column; consequently, the row totals do not add up to 100%.

For example, the optimum cut-off values for semantic dementia were highly selective for semantic dementia, with 100% of the semantic dementia cases factor scores meeting these values (Table 2). Furthermore, there were no misclassifications of patients from other diagnostic groups as semantic dementia. This corresponds to the highest *d*′-value of 4.46 (*P *<* *0.001) for semantic dementia and suggests that the cases with semantic dementia, from which the data-driven diagnostic criteria were derived, show within-group homogeneity and clearly distinct between-group boundaries. This corroborates our earlier qualitative interpretation of semantic dementia as occupying a unique area in the multidimensional space from the unified PCA. Because of the data-driven criteria for other PPA subtypes being less selective for their target subtype, some semantic dementia cases were misclassified; 28.6% of the semantic dementia cases met both the semantic dementia cut-offs and the cut-offs for either mPPA or PNFA.

The data-driven diagnostic criteria for PNFA (*d*′ = 2.03, *P *<* *0.001) were much less selective as not only did they incur misclassifications of all other subtypes, but they also failed to classify all the PNFA cases correctly (hit rates ∼90%). This shows that even the optimal data-driven diagnostic criteria for PNFA were insufficiently selective, implying that PNFA cases do not display within-group homogeneity and distinct between-group boundaries like semantic dementia.

The classification results for lvPPA and mPPA are presented here for completeness, with the caveats that (i) the lvPPA result was derived from a very small sample size and will need validating with a much larger cohort; and (ii) mPPA does not represent an actual subtype category of PPA (instead it represents the label given to cases who do not meet the criteria for any proposed category). This means that the data-driven diagnostic criteria for mPPA would not be expected to have any selectivity for this inherently heterogeneous group. The lvPPA data-driven diagnostic criteria (*d*′ = 3.38, *P *<* *0.001) had a perfect hit rate but some misclassifications of PNFA cases, resulting in a lower *d*′. Consistent with the nature of cases given the mPPA subtype label, the data-driven diagnostic criteria for this group (*d*′ = 2.10, *P *<* *0.001) also showed low selectivity. The mPPA criteria failed to capture all the mPPA cases and incorrectly captured cases from all other subtypes.

## Discussion

This study had two principal aims: (i) to undertake a large-scale direct comparison of PPA and PSA utilizing detailed aphasiological and neuropsychological test batteries; and (ii) to reconsider the phenotype differences across patients with PSA and PPA in terms of graded variations along multiple principal dimensions.

The results confirm that there is meaningful, coherent structure in the language-cognitive variations across PPA and PSA patients. Rather than conceptualizing such variations as mutually exclusive categories, the results indicate that the patients’ variations reflect multiple, continuous, graded dimensions. This alternative approach has multiple advantages: (i) it is able to capture the patterns of overlap between different ‘subtypes’ of PPA and PSA (e.g. overlap in phonological impairments in many PPA and PSA cases) as well as their clear differences; (ii) it captures the variations in performance within each ‘type’ of PPA and PSA; and (iii) it meaningfully situates the ‘mixed’ aphasic patients alongside the other cases to generate a complete clinical picture of PPA and PSA. This is important given that there are high numbers of ‘mixed’ cases in everyday clinical practice. It is, perhaps, important to note that these dimensions are not new categories but rather each patient represents a specific point in the graded multidimensional space.

The cognitive and language impairments in PSA and PPA, both when considered in isolation and when considered in a single unified framework, could be captured by four main dimensions of underlying variation: phonology, semantics, speech production/motor output fluency, and executive-cognitive skill. This finding is a direct replication of what has been found previously for this PSA cohort (Butler *et al.*, 2014; Halai *et al.*, 2017) and by numerous international groups (Kümmerer *et al.*, 2013; Mirman *et al.*, 2015*a*, *b*; Lacey *et al.*, 2017; Tochadse *et al.*, 2018), and found to be statistically stable across different sample sizes and assessment batteries (Halai *et al.*, 2018*a*). These studies have used lesion-symptom mapping methods to show that the principal components are associated with neural correlates that support the labels applied [e.g. components labelled ‘phonology’ having neural correlates in left posterior perisylvian cortical (e.g. superior temporal gyrus) and subcortical regions (e.g. arcuate fasciculus) (Butler *et al.*, 2014; Halai *et al.*, 2017), dorsal parietal white matter (Lacey *et al.*, 2017), which have previously been shown to be involved in phonological processing].

Although outside the scope of this study, neuroimaging information could help to elucidate and further delineate the underlying principal dimensions of variance in both forms of aphasia. Aphasia can be caused by brain injury to cortical but also subcortical brain areas (Naeser *et al.*, 1987, 1989; Hillis *et al.*, 2002, 2004). Therefore, the location of the lesion/atrophy is a critical piece of information to help understand the mapping between behavioural dimensions and neural substrates (Naeser and Palumbo, 1994). For example, situating lvPPA and comprehension-impaired PSA in the same multidimensional space with neuroimaging measures could help to further understand the nature of ‘Wernicke’s area’ (Mesulam *et al.*, 2015). The location of the lesion/atrophy also relates to functional connectivity changes associated with aphasia (Yang *et al.*, 2016; Ranasinghe *et al.*, 2017). Future research could combine the PCA framework used in this study with single- or multimodality imaging information (such as white matter integrity or functional connectivity) to explore how the underlying nature of the brain injury (i.e. abrupt insult versus progressive neurodegeneration) leads to similar/differing neural changes and consequently to similar/differing behavioural symptoms in PSA and PPA; for example, functional connectivity information could explore the possibility of neuronal preservation in PPA compared to PSA (Sonty *et al.*, 2003). Another avenue for future research could be to include longitudinal neuropsychological data in this PCA framework, in order to contrast the temporal profiles of recovery (in a less chronic PSA cohort) versus degeneration. This would inform our understanding of how different aetiologies of brain damage result in a changing aphasic profile in these populations.

The fact that the same underlying dimensions were found for PPA as well as PSA indicates that these dimensions might reflect core ‘primary systems’ for language activities (Patterson and Lambon Ralph, 1999; Ueno *et al.*, 2014; Woollams *et al.*, 2018). Past work has associated these primary systems with different brain areas: phonological processing and working memory with posterior superior temporal lobe and supra-marginal gyrus (Paulesu *et al.*, 1993); semantic representation with anterior temporal lobe (ATL) (Patterson *et al.*, 2007; Lambon Ralph *et al.*, 2017); speech programming and fluency with premotor cortex and key underpinning white matter pathways (Basilakos *et al.*, 2014); and executive functions with frontoparietal networks (Jurado and Rosselli, 2007; Marek and Dosenbach, 2018). As these regions can be affected in both middle cerebral artery PSA (Phan *et al.*, 2005) and PPA (Gorno‐Tempini *et al.*, 2004), the similarity of their phenotypic spectra could reflect varying degrees of impairment to these core primary systems.

Plotting all patients’ factor scores into the shared multidimensional space showed that the non-semantic dementia PPA and PSA cases occupied an almost completely overlapping region of the phonology-semantics space. This contrasts with the semantic dementia cases who occupied an exclusive region of the multidimensional space, signifying their selective semantic impairment in the context of relatively preserved phonological abilities, coupled with motor speech production and executive function that are comparable to healthy control subjects. This might reflect the fact that semantic dementia arises from atrophy in extra-sylvian, ATL regions (Snowden *et al.*, 1989; Hodges *et al.*, 1992; Rosen *et al.*, 2002), whereas the other forms of PPA and PSA are associated with damage to perisylvian cortical and subcortical regions (Hillis *et al.*, 2002, 2004; Grossman and Irwin, 2018).

In the space corresponding to visuo-executive function versus motor speech production, there was separation of the two aetiologies. PSA patients occupied the region signifying less fluent speech production combined with relatively unimpaired visuo-executive ability. Most PPA patients showed the reverse pattern, although some PNFA and mPPA cases showed the combination of poor fluency and poor executive function. This result agrees with previous direct comparisons restricted to the non-fluent subtypes of PSA and PPA (Patterson *et al.*, 2006). This separation is clinically interesting and important as it indicates that certain symptom terms (e.g. fluency) are not used in the same way across patient types; thus, many non-fluent progressive aphasics were more fluent than the ‘fluent’ PSA cases (e.g. anomic and conduction aphasics). This may be relevant for clinical professionals who work with patients with PSA and with patients with PPA; if assessments/tools at their disposal are targeted towards ‘non-fluent’ aphasias then it may be useful to have a formal understanding of how the term ‘fluency’ is applied across PSA and PPA.

The separation in terms of visuo-executive function could reflect an aetiology-driven difference in the neural substrates vulnerable to damage in stroke versus neurodegeneration; cognitive functions supported by regions at the edges of/outside the territory of the middle cerebral artery (MCA) would be less likely to be impaired in PSA than perhaps in some forms of PPA. For example, the multi-demand frontoparietal executive system (Marek and Dosenbach, 2018), and posterior cingulate and other medial regions that support executive function and attention (Jurado and Rosselli, 2007) are situated at the edges/outside of the MCA-perfused regions (Phan *et al.*, 2005), potentially leading to relatively spared visuo-executive function in our PSA cohort.

In addition to facilitating intergroup comparisons, the PCA method revealed graded intragroup differences. The subtypes of non-semantic dementia PPA and PSA occupied only partially differentiated positions within the 4D space, with considerable variation within each ‘subtype’ and overlap of cases across subtypes. This could reflect the overlapping atrophy/lesions in and around the cortical and subcortical perisylvian language regions in these forms of aphasia. Again, these findings indicate that phenotypic variations in non-semantic dementia PPA and PSA are unlikely to reflect different categories but rather graded variations along these dimensions. These graded differences can only be accounted for in categorical classification systems by using ‘mixed’ classifications (Wertz *et al.*, 1984; Sajjadi *et al.*, 2012*a*; Wicklund *et al.*, 2014), but the methods in the current study were able to account for graded variation in a single multidimensional framework comprising four, clinically intuitive underlying dimensions.

Based on this framework, the current diagnostic subtype labels can be reconceptualized as pointers towards particular regions of the multidimensional space, rather than labels for mutually exclusive clinical categories. This approach does not preclude the fact that some labels might be pointers for more exclusive regions of space (e.g. semantic dementia or global PSA) than others (e.g. anomia or PNFA).

In fact, the concept of ‘semantic dementia’ seems to be a uniquely useful pointer for the exclusive region of the multidimensional space occupied by these cases. This aligns with (i) the original descriptions of semantic dementia, in particular the selective nature of their semantic impairment (Warrington, 1975; Snowden *et al.*, 1989; Hodges *et al.*, 1992); and (ii) previous work showing that semantic dementia is distinct from other forms of PPA. Bisenius *et al.* (2017) found that semantic dementia was the most readily differentiable subtype of PPA using support vector machine approaches to evaluate the consensus criteria for PPA. Hoffman *et al.* (2017) applied k-means clustering to behavioural data in PPA and found that of their three-cluster solution, only one cluster was selective for a particular subtype of PPA and this was the semantic dementia cases. This finding agrees with Sajjadi *et al.* (2017), who found that the atrophy patterns for semantic dementia were more easily distinguishable (high sensitivity and specificity) than the other forms of PPA. In our study, by plotting PSA and PPA in the same space we provide support for previous work showing that semantic impairments in semantic dementia are unlike those found in PSA (Jefferies and Lambon Ralph, 2006; Lambon Ralph *et al.*, 2017). This result probably reflects the fact that the distribution of damage in semantic dementia is distinctly different from those in non-semantic dementia PPA and PSA phenotypes. Cases with semantic dementia have hypometabolism and atrophy centred on the ATL bilaterally (Mummery *et al.*, 2000). Data from other methods in healthy participants and patient groups have shown the ATL to be a key region for the formation of coherent concepts (Lambon Ralph *et al.*, 2010). Mesulam *et al.* (2015) showed that the ATL, not regions typically considered to be in ‘Wernicke’s area’, was consistently associated with word comprehension in PPA; this provides further evidence that the ATL is a core component of the language network, despite this region historically being absent from the language network in classic aphasiology. PPA and semantic dementia, in particular, represents a unique platform for investigating the contributions of the ATL to language and semantic processing. In contrast, there is less information to be gleaned about the ATL (other than superior regions) from PSA because most of this region falls outside of the middle cerebral artery territory.

Describing the symptomatology of PSA and PPA in terms of differently graded regions within multidimensional space has a number of potential clinical implications. First, this approach allows us to begin to determine both the range and type of variations that are associated with each of the pre-existing clinical labels, rather than reserving the use of each diagnostic label to a single, invariant prototypical pattern. Second, by extension, it also allows us to establish when and why certain subtypes of PPA are most likely to be confused with each other, and the same for subtypes of PSA. Third, it provides a single unifying framework within which both established and ‘mixed’ aphasias can be considered.

Fourth, future clinical research can explore whether considering the phenotype variations along continuous dimensions (i.e. a transdiagnostic approach) rather than categorical systems might reveal clearer relationships between phenotype and atrophy, pathology or genetic markers. For example, past work in PSA has shown that utilizing raw individual test scores or PSA categories leads to undifferentiated lesion correlates reflecting the whole middle cerebral artery territory rather than specific subregions. When the same lesion-mapping analyses are repeated using the PCA-derived dimensions then much more discrete and interpretable subregions are revealed (*cf*. Butler *et al.*, 2014). These clearer symptom-lesion maps can then be inverted in order to generate lesion-based diagnostics and prediction models (Halai *et al*., 2018*a*). Finally, taking this multidimensional approach could inform a transdiagnostic selection process for treatment, therapy or clinical trials; in order to select a group of patients with relatively homogeneous behavioural symptoms, one could select patients who occupy a shared region of the multidimensional space (thereby sharing symptomatology across the core language systems captured by the dimensions), irrespective of their clinical diagnosis. Indeed, the importance of a transdiagnostic approach has been highlighted for frontotemporal lobar degeneration (FTLD) with regards to shared apathy and impulsivity symptomatology (Lansdall *et al.*, 2017, 2019; Passamonti *et al.*, 2018).

In conclusion, we have shown that the internal structure of variation in PSA and PPA, in isolation or in a unified framework, can be captured with the same four underlying language-cognitive dimensions. Furthermore, semantic dementia appears to represent a robust diagnostic category, whilst patients with other forms of PPA and PSA might be better described in terms of their differently graded positions along these four principal language-cognitive dimensions.

## Supplementary Material

awaa245_Supplementary_Data

## Abbreviations

ATL =: anterior temporal lobe
lv/mPPA =: logopenic/mixed primary progressive aphasia
mPPA =: mixed primary progressive aphasia
PCA =: principal component analysis
PNFA =: progressive non-fluent aphasia
PPA =: primary progressive aphasia
PSA =: post-stroke aphasia
